# Impact of Indoxyl Sulfate on Progenitor Cell-Related Neovascularization of Peripheral Arterial Disease and Post-Angioplasty Thrombosis of Dialysis Vascular Access

**DOI:** 10.3390/toxins9010025

**Published:** 2017-01-07

**Authors:** Chih-Cheng Wu, Szu-Chun Hung, Ko-Lin Kuo, Der-Cherng Tarng

**Affiliations:** 1 Cardiovascular Center, National Taiwan University Hospital, Hsinchu Branch, Hsinchu 30059, Taiwan; chihchengwumd@gmail.com; 2 National Tsing-Hua University, Institute of Biomedical Engineering, Hsinchu 30013, Taiwan; 3 School of Medicine, National Yang-Ming University, Taipei 11217, Taiwan; 4 Division of Nephrology, Taipei Tzu Chi Hospital, Buddhist Tzu Chi Medical Foundation and School of Medicine, Tzu Chi University, Hualien 97004, Taiwan; szuchun.hung@gmail.com (S.-C.H.); kolinkuo8@gmail.com (K.-L.K.); 5 Institutes of Physiology and Clinical Medicine, National Yang-Ming University, Taipei 11217, Taiwan; 6 Division of Nephrology, Department of Medicine, Taipei Veterans General Hospital, Taipei 11217, Taiwan

**Keywords:** chronic kidney disease, dialysis vascular access, indoxyl sulfate, peripheral artery disease, thrombosis, uremic toxin

## Abstract

Patients with chronic kidney disease (CKD) have an increased risk of vascular disease, which is associated with considerable health care costs. Vascular disease in CKD differs clinically and pathobiologically from that in patients with normal renal function. Besides the traditional risk factors, retention of uremic toxins contributes to the pathogenesis of vascular disease in patients with CKD. Indoxyl sulfate is a protein-bound uremic toxin and is inefficiently removed by conventional dialysis. Accumulating evidence suggests that indoxyl sulfate is a vascular toxin involved in atherosclerosis, arteriosclerosis, vascular calcification and vascular repair. Clinically, indoxyl sulfate is associated with total and cardiovascular mortality in patients with CKD. Recent studies have indicated that in addition to coronary and cerebral arteries, indoxyl sulfate plays a role in peripheral artery disease (PAD) and dialysis graft thrombosis. Emerging evidence suggests that indoxyl sulfate is implicated via novel mechanisms, including progenitor cell-related neovascularization and tissue factor-related hypercoagulability. These findings raise the possibility that strategies targeting serum indoxyl sulfate may have the potential to improve the outcomes of PAD and dialysis vascular access in patients with CKD.

## 1. Chronic Kidney Disease and Vascular Disease

### 1.1. Chronic Kidney Disease and Vascular Disease

Compared to patients with preserved kidney function, patients with chronic kidney disease (CKD) are more likely to develop atherosclerotic vascular disease. Vascular disease-related ischemic events cause significant morbidity and mortality in patients with CKD [1]. An increased risk of myocardial infarction and ischemic stroke has been widely reported in patients with CKD [1,2,3,4,5,6]. In addition to cerebral and coronary artery disease, peripheral artery disease (PAD) is highly prevalent among patients with CKD [7]. Creation of vascular accesses for dialysis, including native arteriovenous fistulas (AVFs) and prosthetic arteriovenous grafts (AVGs), is also frequently associated with stenosis and thrombosis [8,9]. Although traditional vascular risk factors are common in patients with CKD, they cannot sufficiently account for the increased vascular events [2]. Understanding the unique pathophysiology of vascular disease in patients with CKD may help to develop strategies for prevention and therapy. In the following sections, we will focus on the novel mechanisms of the effect of indoxyl sulfate on PAD and dialysis vascular accesses.

### 1.2. Peripheral Arterial Disease in Patients with Chronic Kidney Disease

In both the general population and patients with CKD, the risk of PAD increases as the values of glomerular filtration rate (GFR) decline, even after adjustment for possible confounders. The prevalence of PAD increases in patients with CKD, ranging from 7% in Stage 3 to 45% in Stage 5D, which is ten-fold higher than that observed in the general population [7]. Moreover, PAD in patients with CKD presents a unique challenge owing to the poor outcome of revascularization [10]. As a result, the limb amputation rate remains high, and the mortality and morbidity associated with PAD is much higher than that of patients with preserved renal function [6].

### 1.3. Vascular Access Dysfunction in Patients on Hemodialysis

Dysfunction of dialysis vascular accesses continues to be a major source of morbidity and mortality in patients with end-stage renal disease (ESRD). After publication of the dialysis outcome quality initiative guidelines, endovascular interventions have replaced surgical revisions as the primary therapy for dialysis access dysfunction [11]. Although percutaneous transluminal angioplasty (PTA) can achieve a high success rate, recurrent stenosis and thrombosis are usually inevitable. At one year after PTA, only 26%–58% of native fistulas remain functional without subsequent interventions [12]. The outcome of graft accesses is worse than that of native fistulas, as only 40%–50% of AVGs remain functional at six months after intervention. If thrombosis develops in AVGs, the three-month unassisted patency rate ranges from 30% to 40% [13]. As a result, repeated interventions are usually required, placing a substantial financial burden on the health care system [8].

## 2. Vascular Toxicity of Indoxyl Sulfate

Indoxyl sulfate is one of the protein-bound uremic toxins produced by intestinal bacteria as a degradation product of the amino acid tryptophan. Indoxyl sulfate accumulates in CKD, mostly bound to albumin, and is therefore not sufficiently removed by means of conventional dialysis. Increasing number of studies suggest that, in addition to the involvement in the progression of CKD, indoxyl sulfate contributes to the progression of vascular dysfunction.

In clinical studies, indoxyl sulfate is a powerful predictor of overall and cardiovascular mortality in patients with CKD [14,15]. Indoxyl sulfate is positively correlated with aortic calcification and pulse wave velocity [14]. In patients on hemodialysis, indoxyl sulfate is associated with markers related to atherosclerosis, endothelial function, and the incidence of PAD [16,17,18].

Indoxyl sulfate causes endothelial dysfunction in many ways. It increases endothelial oxidative stress via an increase in NADPH oxidase activity and a decrease in intracellular glutathione levels [19]. It causes activation of leukocytes, proliferation of smooth muscle cells, and decrease in nitric oxide availability [20]. These mechanisms are associated with endothelial dysfunction and atherosclerosis progression. Indoxyl sulfate promotes aortic calcification and aortic wall thickening in hypertensive rats, which is manifested by aortic wall thickening and expression of osteoblast-specific proteins [21,22]. Therefore, indoxyl sulfate may be responsible for vascular calcification and arterial stiffness in patients with CKD. Indoxyl sulfate inhibits endothelial proliferation and wound repair, but stimulates the proliferation of vascular smooth muscle cells [23,24,25]. Both the proliferative and antiproliferative effects of indoxyl sulfate lead to abnormal vascular repair and neointima hyperplasia (Table 1).

In addition to the above pathways, pro-thrombosis and angiogenesis have been discovered in recent studies as novel mechanisms by which indoxyl sulfate causes vascular dysfunction. In this review, we focus on the effect of indoxyl sulfate on the angiogenesis of PAD and thrombosis of dialysis vascular accesses.

## 3. Indoxyl Sulfate and Progenitor Cell-Related Neovascularization

### 3.1. Angiogenesis and Peripheral Arterial Disease in Chronic Kidney Disease

PAD in CKD is associated with an increased risk of mortality and morbidity, including limb amputation, and therapy for this condition is challenging [7]. Progression of atherosclerosis, which is accelerated by uremic state, may be the main cause of poor outcome in patients with CKD. Endothelial dysfunction is an early marker of atherosclerosis. In CKD, oxidative stress and nitric oxide (NO) deficiency play an important role in endothelial dysfunction. Tumur et al. have shown that indoxyl sulfate inhibits endothelial cell viability by inhibition of NO production [38]. Several studies described that indoxyl sulfate upregulates the expression of adhesion molecules and enhances leukocyte–endothelial cell interactions [20,33,34]. These findings suggest that indoxyl sulfate may contribute to the progression of atherosclerosis in patients with CKD by inducing inflammation and endothelial dysfunction.

Neovascularization, defined as the sprouting of new blood vessels from pre-existing vascular structures, is an essential physiological process to cope with tissue ischemia. The ability of collateral vessel formation in ischemic tissues is decreased in patients with CKD [39]. In a rat model, Jacobi et al. showed impairment of neovascularization owing to increased free radical production in CKD [40]. Neovascularization involves the proliferation of both local endothelial cells and circulating endothelial progenitor cells (EPCs) derived from the bone marrow [41]. EPCs have been reported to improve angiogenesis and wound healing [42]. Patients with advanced CKD show a decline in the number and function of EPCs [43]. However, information regarding the effect of indoxyl sulfate on EPC-mediated neovascularization is scarce.

### 3.2. Mechanisms Underlying the Effect of Indoxyl Sulfate on Neovascularization

Indoxyl sulfate has been shown to have direct effects on EPCs through NO-dependent pathways both in vitro and in vivo. In cultured EPCs, indoxyl sulfate decreased the expression of phosphorylated endothelial NO synthase and vascular cell adhesion molecule-1, and increased reactive oxygen species. These effects led to decreased homing capacity and proliferative capacity, increased senescence and autophagy, as well as decreased migration and angiogenesis of EPCs [36].

Indoxyl sulfate also suppresses the angiogenic function of EPCs by inhibiting hypoxia-induced HIF-1α activation and consecutive interleukin (IL)-10 and VEGF synthesis [37]. In vitro, indoxyl sulfate inhibited hypoxia-induced EPC migration and tube formation. These effects could be mitigated by the addition of IL-10 and VEGF. In human EPCs, indoxyl sulfate suppressed the up-regulation of VEGF, IL-10, and HIF-1α. Experiments with HIF-1α siRNA and an IL-10 inhibitor demonstrated that an increase in HIF-1α and, consequently, in IL-10 increase is necessary to enhance VEGF levels.

Accumulating evidence shows that the cardiovascular effect of indoxyl sulfate is mediated by activation of the aryl hydrocarbon receptor (AHR) [29,44]. AHR requires aryl hydrocarbon receptor nuclear translocator (ARNT) to regulate gene expression. ARNT, also called HIF-1β, is required by HIF-1α to enhance gene expression in response to hypoxia. Indoxyl sulfate is an endogenous ligand of AHR. Activation of AHR by indoxyl sulfate suppresses the accumulation of the HIF-1α-ARNT complex in the nucleus in inverse proportion to the increase in the nuclear AhR-ARNT complex, resulting in inhibition of HIF-1α activity [45].

**Figure 1:**
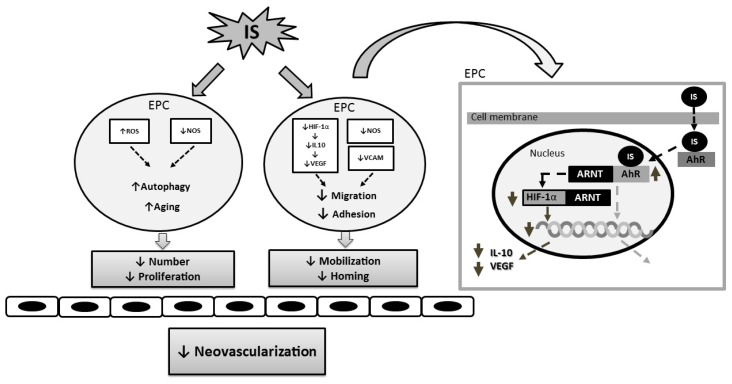
Mechanisms of the effect of indoxyl sulfate on neovascularization in patients with kidney disease (AhR, aryl hydrocarbon receptor; ARNT, aryl hydrocarbon receptor nuclear translocator; EPC, endothelial progenitor cell; IS, indoxyl sulfate, NOS, nitric oxide synthase; ROS, reactive oxygen species; HIF, hypoxic induced factor; IL-10, interleukin-10; VCAM, vascular cell adhesion molecule; VEGF, vascular endothelial growth factor).

The effect of indoxyl sulfate on neovascularization in ischemic hindlimbs has been demonstrated in an animal model [37]. Subtotal nephrectomy was performed in eight-week-old male C57BL/6 mice, which were then divided into three groups, namely the vehicle group, the indole group, and the indole plus AST-120 group. Eight weeks later, unilateral hindlimb ischemia (HI) surgery was performed on all animals. At Week 2 and Week 4 after HI surgery, the blood flow recovery was significantly impeded in the indole group compared with the vehicle group, but was improved in the indole plus AST-120 group. In parallel, compared with the vehicle group, circulatory Sca1/Flk-1-positive EPCs and the density of CD31^+^ capillary neovessels in the muscle of ischemic limbs were significantly reduced in the indole group. Similarly, the reductions were significantly reversed in the indole plus AST-120 group. Our results support the concept that indoxyl sulfate may be a therapeutic target for the progenitor cell-related neovascularization of ischemic tissue. The likely mechanisms underlying the effect of indoxyl sulfate on EPCs and neovascularization are summarized in Figure 1.

## 4. Indoxyl Sulfate and Thrombosis of Dialysis Vascular Accesses

### 4.1. Thrombosis of Dialysis Vascular Accesses

Thrombosis is the most common cause of secondary vascular access failure [46]. According to Virchow’s triad, vascular thrombosis involves an interplay among blood stasis, vessel injury, and hypercoagulability [47]. A major predisposing factor for thrombosis of vascular accesses is stenoses in the outflow veins, which cause blood stasis and are noted in 60%–80% of the cases. Nonetheless, 20%–40% of the cases still occur in the absence of stenosis. In addition, not all vascular accesses with stenoses experience thrombosis; thus, there must be other factors contributing to thrombosis. For example, low-flow state secondary to hypotension has been proposed to precipitate thrombosis [48]. Other causes, such as vascular injury, inflammation, and inadequate hemostasis of dialysis accesses, may also precipitate the development of vascular access thrombosis [49]. Previous studies have demonstrated a prothrombotic state in patients on hemodialysis [1,2,50,51,52,53]. Both inherited and acquired thrombophilia have been reported as possible causes of dialysis access thrombosis [54,55,56]. Nonetheless, neither antiplatelet drugs nor anticoagulants have a significant effect.

Recently, indoxyl sulfate was implicated in the increased risk of thrombosis in patients on hemodialysis. In a study on 100 patients on hemodialysis without vascular access dysfunction, baseline indoxyl sulfate levels were linked to the number of vascular access thrombectomies over three years [18]. In another study on 306 patients with vascular access dysfunction, baseline serum indoxyl sulfate levels were associated with thrombosis of vascular accesses after endovascular interventions [57]. A total of 175 patients with graft accesses and 131 patients with native accesses were enrolled. After a median follow-up of 32 months, indoxyl sulfate levels independently predicted thrombotic events of vascular accesses only in patients with graft accesses. No association between indoxyl sulfate and restenosis was found. These clinical studies suggest that indoxyl sulfate may be involved in the thrombotic events of dialysis vascular accesses. Nonetheless, animal models or therapeutic trials are required to validate these observational findings.

### 4.2. Mechanisms Underlying the Effect of Indoxyl Sulfate on Thrombosis

Blood stasis secondary to stenoses is the most common mechanism for thrombosis of dialysis vascular accesses. Nonetheless, the role of indoxyl sulfate in stenoses of dialysis vascular accesses remains unclear. Indoxyl sulfate has been demonstrated to play a critical role in renal fibrosis, mediated by inflammatory reactions and pro-fibrotic cytokines [58]. Nonetheless, both proliferative and antiproliferative effects of indoxyl sulfate on smooth muscle cells have been reported in vitro [14,59,60]. In a hypertensive rat model, indoxyl sulfate induced cell cycle inhibitors and caused cell senescence in the calcification area of arcuate aorta [60]. The net effect of indoxyl sulfate on the development of intimal hyperplasia still needs to be determined.

In recent years, a growing body of evidence showed that indoxyl sulfate increases the tissue factor in endothelial cells and vascular smooth muscle cells [32,33]. Tissue factor is a crucial mediator of vascular thrombosis. Using a de-endothelialized, postinterventional model, Chitalia et al. exposed human vascular smooth muscle cells to coronary-like blood flow. These smooth muscle cells were pretreated with uremic serum obtained from patients with ESRD. Uremic serum significantly upregulated the tissue factor in vascular smooth muscle cells [32]. In a subsequent study, they showed that indoxyl sulfate regulated tissue factor stability through the AHR signaling pathway, and that AHR was an effective antithrombotic target [44]. Circulating tissue factor was elevated in patients with CKD and was positively correlated with plasma indoxyl sulfate levels [33]. The indolic uremic solutes increased the production of tissue factors by AHR activation in endothelial cells, evoking a “dioxin-like” effect. These findings suggest that indoxyl sulfate and AHR may comprise a promising therapeutic platform to treat or prevent thrombosis in patients with CKD [34].

**Figure 2:**
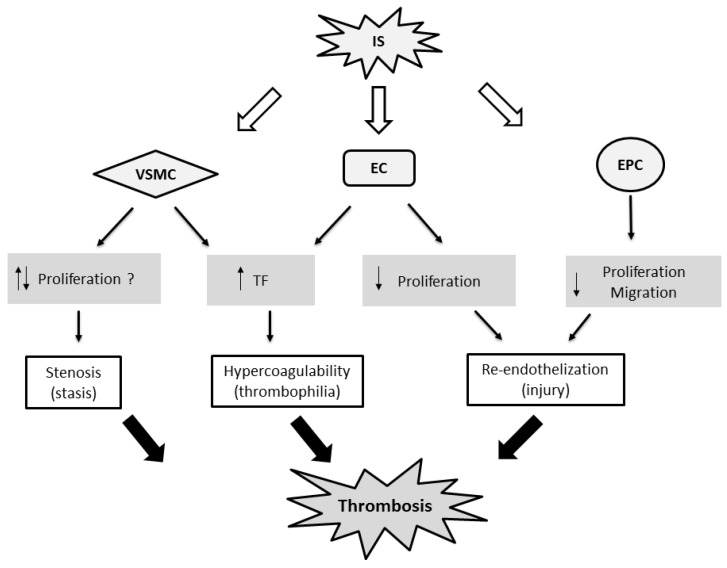
Possible mechanisms of indoxyl sulfate induction of vascular thrombosis. EC, endothelial cell; EPC, endothelial progenitor cell; IS, indoxyl sulfate, TF, tissue factor; VSMC, vascular smooth muscle cell.

An intact endothelium possesses both anti-platelet and anti-thrombotic properties. Exposure of the subendothelial matrix is highly thrombogenic. Therefore, re-endothelialization may be another critical factor related to thrombosis after endovascular therapy. Indoxyl sulfate inhibits endothelial proliferation and impairs wound repair in vitro [43]. Endothelial progenitor cells have been shown to mobilize and incorporate into the denuded vessel wall, a process associated with accelerated re-endothelialization [61]. Clinical studies have shown progenitor cell deficiency to be associated with restenosis and thrombosis after endovascular intervention [62,63,64]. Indoxyl sulfate has a deleterious effect on progenitor cell function, which may precipitate thrombosis through delayed re-endothelialization [36]. Nonetheless, the link among indoxyl sulfate, re-endothelialization and thrombosis still remains unclear. The possible pro-thrombotic mechanisms of indoxyl sulfate are summarized in Figure 2.

## 5. Overall Summary

Traditionally, indoxyl sulfate is considered a vascular toxin involved in atherosclerosis, arteriosclerosis, vascular calcification and vascular repair in patients with CKD. Recent studies have shown that different mechanisms are involved in indoxyl sulfate-related vascular toxicity, including progenitor cell-related neovascularization in PAD and tissue factor-related hypercoagulability in AVGs. Further studies are warranted to validate these novel mechanisms and to explore their clinical applications in patients with CKD.

## Figures and Tables

**Table 1:** Primary effect of indoxyl sulfate on the different types of cells involved in vascular dysfunction of chronic kidney disease.

Cells	Primary Effect	Reference
Endothelial cell	Induction of ROS	Dou [26], Yu [17], Itoh [27]
	Inhibit endothelial NO production	Yu [17]
	Increase endothelial microparticle release	Faure [28]
	Increase tissue factor production	Gondouin [29]
	Inhibit endothelial cell proliferation	Dou, Yu [17]
	Inhibit endothelial cell migration	Kharait [30]
Smooth muscle cell	Increase proliferation	Yamamoto [24]
	Inhibit proliferation	Mozar [31]
	Reduce tissue factor breakdown	Chitalia [32]
Leukocyte	Increase leukocyte adhesion	Ito [33], Tumur [20], Pletinck [34]
	Increase inflammatory cytokine expression	Lekawanvijit [35]
Progenitor cell	Induction of ROS	Wu [36]
	Inhibit NO production	Wu [36]
	Inhibit HIF/IL-10/VEGF pathway	Hung [37]
	Decrease in number and function	Hung [37], Wu [36]

HIF, hypoxia-inducible factor; NO, nitric oxide; ROS, reactive oxygen species; VEGF, vascular endothelial growth factor.
